# Investigating the Pathological Relevance of N-acylsphingosine Amidohydrolase 2 (ASAH2) and Related Proteins in Alzheimer’s Disease

**DOI:** 10.7759/cureus.87463

**Published:** 2025-07-07

**Authors:** Abdalla Khabazeh, Eunju Cho, Victor Ekuta, Jetish Kumar, Nastaran Poursahdi, Timothy Wong, Pengfei Wu, Gewei Lian, Towia Libermann, Volney Sheen

**Affiliations:** 1 Neurology, Beth Israel Deaconess Medical Center, Harvard Medical School, Boston, USA

**Keywords:** alzheimer’s, asah2, biomarker, neurodegenerative, proteins, vesicles

## Abstract

Background

Alzheimer’s disease (AD) is a progressive neurodegenerative disorder mainly characterized by progressive cognitive decline and memory loss. Identifying candidate biomarkers before the clinical onset of AD is crucial for enabling earlier diagnosis and timely therapeutic intervention. Among different molecular targets, N-acylsphingosine amidohydrolase 2 (ASAH2), a key enzyme in ceramide metabolism, has been linked to many neurodegenerative diseases, including AD. This study investigates ASAH2 expression in human serum and AD mouse models to explore its potential as an early biomarker and to understand its involvement in AD progression.

Methods

The protein levels in the serum of pre-AD, mild cognitive impairment, and control patients were measured using the SOMAscan assay platform (SomaLogic Inc., CO, USA) to identify potential candidate biomarkers for preclinical AD. ASAH2 expression at different Alzheimer's stages of triple transgenic (3x-TG) Alzheimer's mice organs and tissues was analyzed. Human serums and hepatoblastoma cells (also referred to as HepG2 cells) were stained and quantified with ASAH2, membrane, and vesicle trafficking proteins.

Results

We found that neutral ceramidase (ASAH2/ASAH2B) and sortilin-related VPS10 domain-containing receptor 2 (SORCS2) were significantly elevated in the serum of individuals who later developed Alzheimer's dementia. Similarly, 3x-TG AD mice showed an increased ASAH2 expression at three months, followed by a marked decline at 14 months compared to age-matched non-3x-TG controls. In non-3x-TG mice, ASAH2 was highly expressed in visceral organs such as the heart, liver, kidneys, lungs, and stomach, but was nearly absent in the brain. Ingenuity pathway analysis revealed dysregulation in lipid trafficking and inflammatory pathways. Additionally, we observed elevated lysosomal-associated membrane protein 1 (LAMP1) and reduced levels of filamin A and RAB7 (a member of the RAS oncogene family) in the pre-AD mild cognitive impairment group.

Conclusion

Our findings suggest that ASAH2 may play a significant role in the pathogenesis of AD, potentially contributing to early molecular changes that precede clinical symptoms. In addition, the identification of a subgroup of lipid- and membrane-associated proteins provides promising candidates for predictive biomarkers that could facilitate earlier diagnosis and offer new insights into the mechanisms underlying disease progression and possible therapeutic targets for AD prevention.

## Introduction

Alzheimer's disease (AD) is a progressive neurodegenerative disease that causes dementia in many elderly people and in some individuals with Down syndrome (DS). To date, no cure has been found [1], and more than two-thirds of individuals aged 65 or older diagnosed with dementia are identified as having AD. AD can be hereditary, following an autosomal dominant pattern, and is associated with mutations in three specific genes: the AAP gene located on chromosome 21, presenilin 1 (PSEN1) found on chromosome 14, and presenilin 2 (PSEN2) situated on chromosome 1. Furthermore, the presence of the APOE e4 allele and Apolipoprotein E, a genetic marker, also plays a crucial role as a risk factor for the development of AD. Interestingly, some forms of AD, whether occurring within families or sporadically, have been linked to variations in the gene responsible for encoding the sortilin receptor (SORT1) [2]. Clinically, the diagnosis of AD was mainly based on the patient and family history, neurological examination, and cognitive testing. If appropriate, functional imaging by positron emission tomography (PET) imaging of brain beta-amyloid (Abeta) deposition and measurement of Abeta in cerebrospinal fluid at present is the best-characterized method for AD diagnosis. Their utility, however, is limited by invasiveness, cost, and restricted availability. AD can be confirmed postmortem by demonstrating the hallmark findings of amyloid plaques and neurofibrillary tangles in the brain [3-5].

The growing recognition that the disease process is ongoing, damaging the brain long before clinical findings appear, has reinforced the need for biomarkers that allow for early diagnosis and objective assessment of clinical responses to putative treatments. Blood-based biomarkers would provide a less invasive and potentially more cost-effective means for early detection and monitoring of AD progression. Numerous studies have identified a subgroup of plasma proteins that correlate with Abeta burden and could serve as biomarkers [6-13]. Recent work has also shown the ability to measure Abeta and phospho-tau 217 in blood and correlate such levels with AD progression [10,11,14,15].

To identify other potential candidate biomarkers for preclinical AD and gain insights into pathophysiology, we performed SOMAscan (SomaLogic Inc., CO, USA) profiling studies using samples from individuals with mild cognitive impairment (MCI) that progressed later to AD in the Ginkgo Evaluation of Memory (GEM) study. We found elevated levels of neutral ceramidase (N-acylsphingosine amidohydrolase 2 (ASAH2)/ASAH2B) in the serum of these individuals. This is a membrane-bound neutral ceramidase that hydrolyzes ceramide into sphingosine and fatty acids, regulating sphingolipid metabolism. The protein modulates key cellular processes such as apoptosis and inflammation, impacting neuronal survival. Dysregulation of ASAH2 has been implicated in neurodegenerative diseases, including AD, Parkinson’s disease, and Huntington’s disease. In AD, altered ASAH2 expression disrupts lipid signaling, contributing to synaptic loss and cognitive decline. These changes support its potential as a biomarker for early detection and progression monitoring.

Prior studies show decreased expression of ASAH2 in end-stage AD [16]. We identified a similar progression of high to low ASAH2 expression in the triple transgenic mice. ASAH2 has high expression in visceral organs and minimal expression in the brain. Given prior studies suggesting that ASAH2 is an integral membrane protein, reportedly found in hepatic endosomes, and associated with caveolin [17,18], we examined the expression of other vesicle membrane-associated proteins in the pre-AD serum samples. We found decreased FLNA and RAB7 and increased lysosomal-associated membrane protein 1 (LAMP1) levels. Collectively, these findings suggest that changes in a subgroup of membrane-associated vesicle proteins might provide predictive value for AD onset and insight into neuropathology.

## Materials and methods

Human subjects

The study was conducted at Beth Israel Deaconess Medical Center, Harvard Medical School, Boston. Beth Israel Deaconess Medical Center (BIDMC) committee and in affiliation with Harvard University issued approval 2017D000148. We obtained samples from individuals enrolled previously in the GEM study from the National Centralized Repository for Alzheimer's Disease and Related Dementias (NCRAD). Five subjects developed AD after five years (average time to dementia 4.2 years), but were asymptomatic at the time of blood draw; five healthy controls, and five individuals with MCI who did not progress to AD over the duration of the study. Two or three females/males were used in each group. No significant age differences (p<0.05) were observed in the different cohorts. 

SOMAscan proteomic assay and analysis

Serum from individual subjects is profiled using a SOMAscan assay (SomaLogic Inc., CO, USA), which was specifically developed for assessment of secreted proteins, assessing over 1,300 proteins. Around 150 μl of serum per patient is used to measure all proteins in a multiplex manner. Each aptamer targets a specific protein and is tagged with a unique DNA sequence, permitting its quantification. Protein-aptamer levels are quantified on Agilent hybridization arrays (Agilent Technologies, Inc., CA, USA) in relative fluorescent units and analyzed using SomaSuite version 1.0 (SomaLogic Inc.). Protein levels from 20 shared test subjects are used to normalize the data across individual assay runs. An additional 221 age-matched healthy control subjects from prior SOMAscan assay studies were included in this study for validation. 

Each of the 150 ml serum samples was used to measure all proteins, up to 4,000 proteins, in a multiplex manner by SomaLogic Inc. Briefly, each aptamer targets a specific protein and is tagged with a unique DNA sequence, permitting its quantification. Protein-aptamer levels were quantified on an Agilent hybridization array in relative fluorescent units and analyzed using SomaSuit version 1.0 (SomaLogic Inc.). Protein levels from 20 shared test subjects were used to normalize the data across individual assay runs.

For analysis, we used the ANOVA test and Tukey's multiple comparison results to determine the significance of any potential protein candidates identified (i.e., ASAH2/ASAH2B, sortilin-related VPS10 domain-containing receptor 2 (SORCS2)) through the SOMAscan platform and determine the subset of biomarkers that collectively provide predictive value for AD progression and assess the optimal receiver operating characteristic (ROC) threshold. To provide insight into potential mechanisms, data sets for Ingenuity Pathway Analyses (IPA) are analyzed by a pairwise comparison and the Wilcoxon signed-rank test to derive biologically meaningful results, as per modifications of our previously published work. The IPA is a curated database of previously published findings on mammalian biology from public literature (Ingenuity Systems, Germany). Reports on individual studies of genes or proteins in humans, mice, or rats are first identified from peer-reviewed publications, and findings are then encoded into an ontology by content and modeling experts. Manual extraction and curation probably result in more specific and comprehensive interactions, with much fewer false positives than automated alternatives. The process involves the following steps: Proteins identified as significant from the experimental data sets are overlaid onto the interactome. Focus proteins are identified as the subset having direct interaction(s) with other proteins in the database. The specificity of connections for each focus protein is calculated by the percentage of its connections to other significant proteins. The initiation and growth of pathways proceed from proteins with the highest specificity of connections. Each pathway has a maximum of 40 proteins. Pathways of highly interconnected genes are identified by statistical likelihood using equations as previously described. 

Study cohort and sample selection

Serum samples were obtained from participants of the GEMS, a randomized, double-blind, placebo-controlled clinical trial aimed at evaluating the efficacy of Ginkgo biloba in preventing dementia. Participants were community-dwelling individuals aged 75 to 96 years, recruited from four U.S. communities: Hagerstown, MD; Pittsburgh, PA; Winston-Salem/Greensboro, NC; and Sacramento, CA. The inclusion criteria required participants to be within the specified age range and to provide informed consent. Exclusion criteria encompassed the presence of neurological or neurodegenerative diseases, a higher risk of dementia, current use of cognitive enhancers or treatments for AD, unwillingness to restrict vitamin E intake, use of over-the-counter Ginkgo biloba, bleeding disorders, thrombocytopenia, or the use of anticoagulants or other medications posing similar risks.

Cell culture

Human hepatocellular carcinoma (HepG2) and human embryonic kidney 293 (HEK293) cells were maintained in Dulbecco’s modified Eagle Medium (DMEM) supplemented with 10% fetal bovine serum (FBS) and 1% penicillin/streptomycin (P/S; maintenance medium) in a humidified incubator at 37°C with 5% CO2. The culture medium was replaced every two to three days. For HEK293 cells, the cells were transfected with the pcDNA3-GFP-ASAH2 construct for six hours before the experiment.

Animals

From the Jackson Laboratory, 3xTg AD mice (033930) expressing APPSwe and tauP301L with Psen1 PS1M146V mutation were purchased and maintained on a B6/129 genetic background. All mice were housed and provided with water and food on a 12-hour light/dark cycle according to animal protection regulations. In this study, E17, three-, nine-, and 14-month-old heterogeneous (female and male) and age-matched non-transgenic (NonTg) mice were used. All animal experimental procedures were approved by the Institutional Animal Care and Use Committee of Beth Israel Deaconess Medical Center (BIDMC) and in affiliation with Harvard University (IACUC 056-2022). The genotype of 3xTg AD mice was confirmed using real-time polymerase chain reaction (PCR) analysis. The primer sequences are provided by the Jackson Laboratory genotyping protocols database.

Western blot assay

The animal organs and the HepG2 cells were homogenized and prepared as previously described for Western blot analysis [19]. Briefly, the whole brain, liver, and other organs were collected after E17 or six-month-old mice were anesthetized by intraperitoneal administration of 35 mg/kg pentobarbital sodium (1%). The skull of each animal was exposed via sagittal cutting and fascia reflection at the scalp midline. A 3.0-mm-diameter craniotomy on the right hemisphere was made between the lambda and bregma and 2.0 mm from the sagittal suture. A 2.5-mm-diameter pillar was positioned at the hit site to release a 30 g weight dropping from 10 cm high along a stainless-steel string and then stricken after the skull flap was removed. The cell lysates and organ samples were prepared by homogenization with Pierceä RIPA buffer (Thermo Scientific Inc., MA, USA), protease inhibitor cocktail (Sigma, MilliporeSigma, MA, USA), and phosphatase inhibitor cocktail (Sigma). The homogenized samples were centrifuged at 13,200 rpm for 20 min, and the supernatant was collected to evaluate the amount of ASAH2 (Abclonal Technology, China, A12596), Filamin A (FLNA, FlnA; Abcam, MA, USA, ab76289), RAB7 (Abcam, ab50533), and LAMP1 (Abcam, ab25630) by Western blot.

Immunohistochemistry (IHC)

The fixed brain and liver tissues were rinsed with 1x phosphate-buffered saline (PBS) three times and incubated with 0.5% Triton X-100 (Sigma) permeabilization solution in 1x PBS at room temperature for 30 min. Thereafter, the sections were incubated with ASAH2 (Abclonal, A12596) in 3% non-fat dry milk as a primary antibody at 4°C overnight. The next day, the tissues were washed with 1x PBS, and the ImmPRESS® Universal PLUS Polymer Kit Peroxidase kit (Vector Laboratories, CA, USA, MP-7800) was used for the secondary antibody. For the counterstain, we used only one stain, hematoxylin, specifically Gill 3 Stain, as H&E both stains could have obscured the results. For immunocytochemistry, HepG2 and HEK293 cells were fixed with ice-cold 5% trichloroacetic acid (TCA) at room temperature. After 10 min, the cells were rinsed with PBS three times, followed by permeabilization solution (0.02% saponin) and 0.5% bovine serum albumin (BSA) at room temperature for 30 min. Thereafter, the cells were washed and incubated overnight at 4°C with the primary antibody: ASAH2 (Abclonal, A12596), BIG2 (Santa Cruz Biotechnology, CA, USA, sc-398042), Filamin A (FLNA, FlnA; Abcam, ab76289), Caveolin-1 (CAV-1; BD Transduction Laboratories, NJ, USA, 610406), RAB7 (Abcam, ab50533), and LAMP1 (Invitrogen, Thermo Fisher Scientific, MA1-164). The next day, fluorescence-conjugated secondary antibody Alexa Fluor 488 or Alexa Fluor 594 was used to stain the cells at room temperature for one hour. After washing three times, the images obtained from a confocal microscope (Leica, Leica Microsystems GmbH, Germany) were analyzed using ImageJ software (National Institutes of Health (NIH), USA).

Statistical analysis

All data were expressed as mean ± standard error of the mean. Statistical analysis was performed with GraphPad Prism (version 8.4.2; GraphPad Software, La Jolla, CA, USA), and statistical significance was set at p<0.05. Western blot data were analyzed using Student’s T-test (two-tailed) or one-way analysis of variance (ANOVA), followed by Tukey’s multiple comparison test to determine statistically significant differences.

## Results

Elevation of ASAH2 expression in the pre-AD stage of human serum

**Figure 1:**
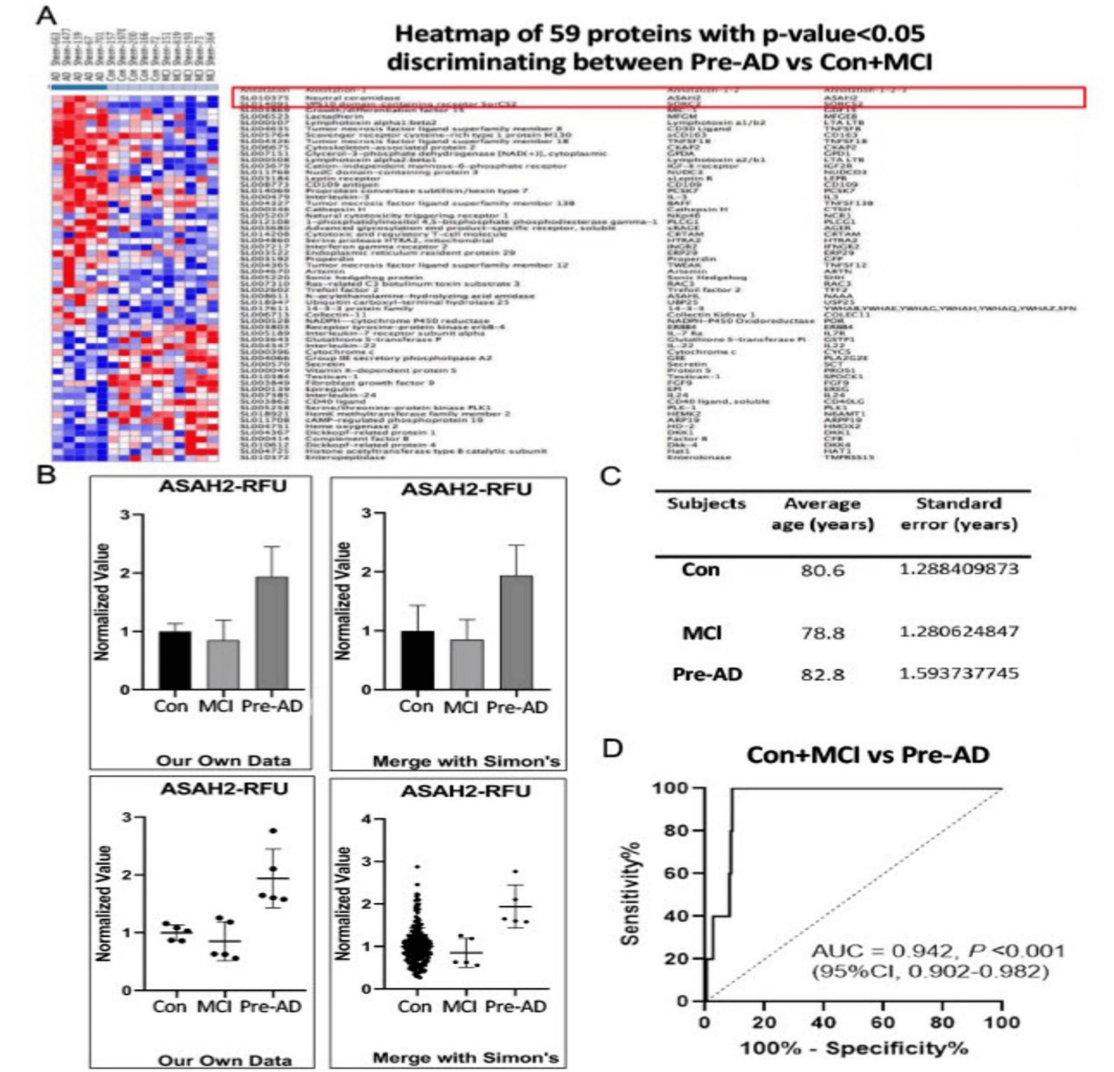
Protein expression profiling of AD patient-derived serum by SOMAscan assay. A: Heatmap demonstrates the significantly changed protein expressions by SOMAScan assay within the pre-AD population compared to the MCI and age-matched Con groups. The red color represents the upregulated proteins, whereas blue represents the downregulated ones, p<0.05. B: The quantification of ASAH2/ASAH2B protein levels from MCI, pre-AD, and Con human serum samples. An additional 221 age-matched subject serums were included in the Con group (right), p**<0.0001. C: Table provides the average age between individuals in each group. n=226 serum samples for Con, n=5 serum samples for each of the MCI and pre-AD groups. D: The receiver operating characteristic (ROC) curve shows an optimal ROC threshold of 1.559, yielding a sensitivity of diagnosis at ~100% and a misdiagnosis rate of <10%. con: control; MCI: mild cognitive impairment; pre-AD: pre-Alzheimer’s disease; RFU: relative fluorescence unit; AUC: area under the ROC curve; ASAH2: N-acylsphingosine amidohydrolase 2

We measured protein levels in serum using the SOMAscan assay, which is an aptamer-based assay that allows for measurement and quantification of 1300 proteins (in the version used here). We identified 59 candidate proteins that showed significant protein expression differences (p<0.05) between AD and both healthy control + MCI groups (Figure 1A). Two of the candidate proteins (ASAH2/ASAH2B) and SORCS2 were of particular interest, as their expression levels in the pre-AD population did not overlap with either the MCI or the healthy control groups. 

To offset the potential confounding variable of a limited sample size, we further evaluated expression levels in the AD group by comparison with a previous data set of age-matched healthy controls. We compared healthy controls from an additional 221 serum samples (average age 76.4, SE 2.438 years). The relative fluorescence units (RFUs) comparing the mean values and standard deviations, as well as values for each individual subject, are graphically following comparison of the initial screen and then including the additional control subjects (Figures 1B, 1C). Statistically, the ANOVA test and Tukey's multiple comparison results (with additional healthy control results included) show the respective p-values: healthy control and MCI versus pre-AD (p<0.0001), healthy control versus MCI (p = 0.7254), healthy control versus pre-AD (p<0.0001), and MCI versus pre-AD (p = 0.0003). 

To assess whether a certain threshold of ASAH2/ASAH2B serum level indicates a higher risk of developing clinical AD, we calculated a ROC curve. The ROC determines the diagnostic ability of a binary classifier system as its discrimination threshold varies (Figure 1D). After normalization, the lowest value in the pre-AD group shows a relative RFU level of 1.5797. Of the 226 healthy control individuals, only 21 samples have a level above 1.57. The ROC threshold with the best specificity and sensitivity is 1.559. Such a clinical threshold yields a sensitivity of ~100% with a misdiagnosis rate of <10%.

Expression of ASAH2 in the different organs of embryonic and adult mice

**Figure 2:**
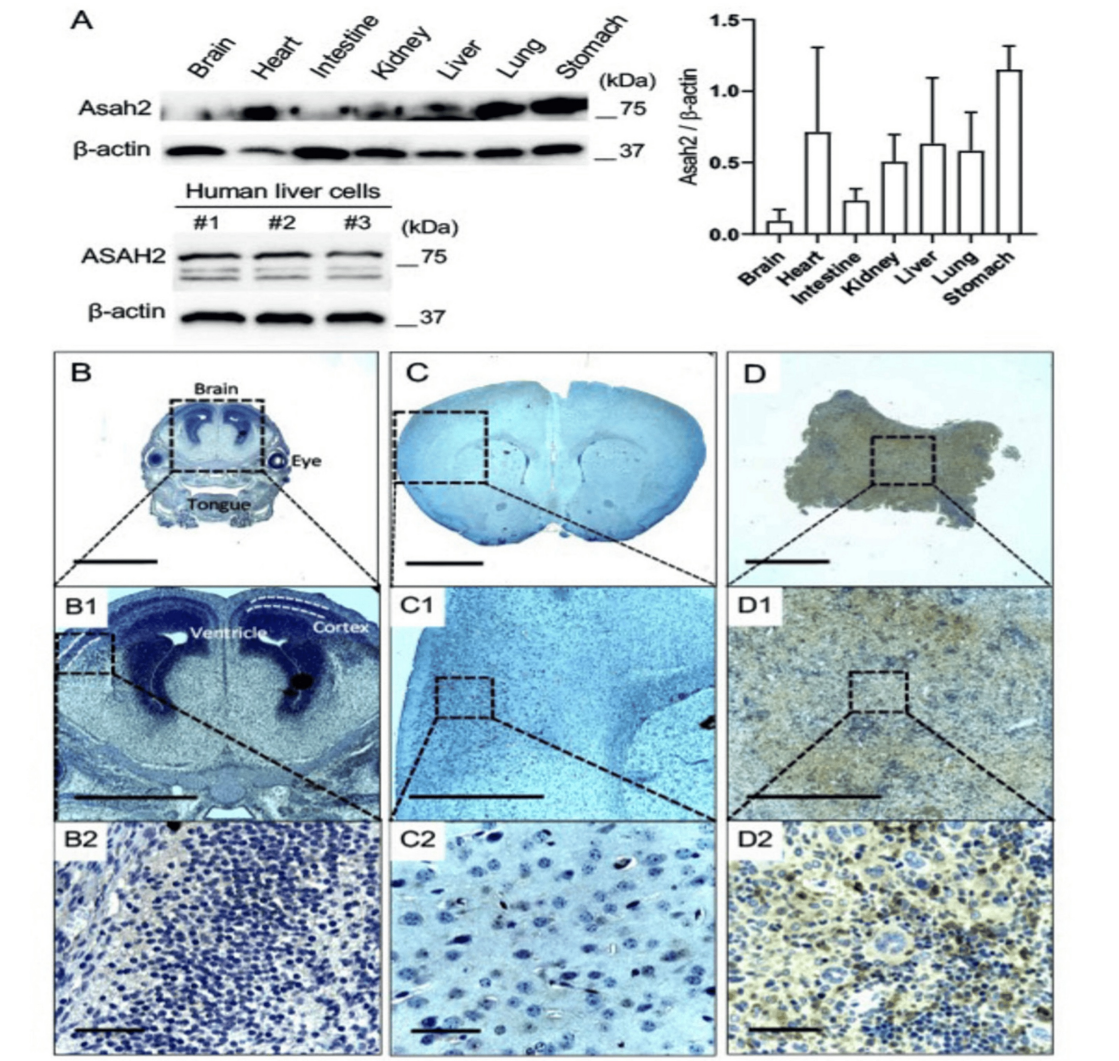
The comparison of ASAH2 protein expression in embryonic and adult mice tissues and organs. A: Western blot analyses of N-acylsphingosine amidohydrolase 2 (ASAH2) protein expression in six-month-old non-transgenic (NonTg) mice organs (above) and HepG2 human liver cells (below). Increased expression is seen in the visceral organs, with significantly reduced expression in the brain. This experiment was independently repeated four times. ASAH2 protein expression in the liver was compared with that of the brain (p=0.114), heart (p=0.885), intestine (p=0.885), kidney (p=0.885), lung (p=0.885), and stomach (p=0.342) (n=4 samples per group). Student’s t-test (unpaired). B-D: Brightfield photomicrographs of immunostaining against ASAH2 and counterstained with hematoxylin and eosin on non-Tg mice brains at various ages. Minimal ASAH2 protein expression is seen in either the E17 or adult-age mouse brain compared to the adult liver (shown in (D)). Scale bars, 2 mm (B-D), 1 mm (B1-D1), 50 mm (B2-D2).

Given that ASAH2 was detected in human serum, we asked where the protein might show the greatest levels of expression in various organ systems. Western blot analyses demonstrated robust expression of ASAH2 protein in six-month-old adult non-Tg mice liver, heart, and gut (Figure 2A upper). Expression in the human liver was further confirmed by a Western blot of human HepG2 hepatoma cells (Figure 2A below). Surprisingly, very little expression was seen in the brains of adult or E17 mice by either Western blot or immunohistochemistry (Figures 2B, 2C). In the adult mouse liver, ASAH2 is strongly expressed in a subset of hepatocytes (Figure 2D).

Decreased ASAH2 expression in the end-stage AD of 3xTg AD mice liver

**Figure 3:**
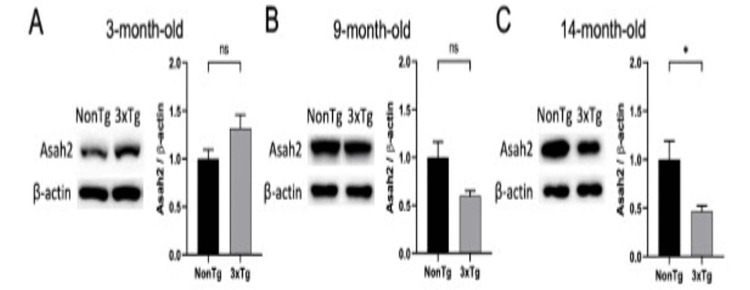
ASAH2 protein levels in the stage-dependent disease progression of the 3xTg AD-like animal model liver. Western blot analyses of N-acylsphingosine amidohydrolase 2 (ASAH2) protein expression levels in non-transgenic (non-Tg) and 3xTg mice liver. Quantification is shown graphically to the right. A: A trend toward increased levels of ASAH2 protein is seen in three-month-old 3xTg AD mice compared to NonTg mice. B, C: A trend toward decreased levels of ASAH2 protein is seen in nine-month-old 3xTg AD mice, which reaches statistical significance by 14 months in 3xTg AD mice compared to NonTg mice. n = >= 4 mice per group. *p<0.05 (C), Student’s t-test (unpaired).

To assess whether similar changes in ASAH2 from human samples were seen in AD mice models, we analyzed the expression of this protein in the liver of the 3xTg mouse AD model by Western blot (Figure 3). The liver was chosen given high levels of ASAH2 expression and prior strong linkage of AD with liver dysfunction [20]. Aligned with the human serum results, the ASAH2 protein expression in 3xTg AD liver showed a trend toward increased levels in the early stage (three-month-old) compared to NonTg (Figure 3A). A decline in levels of ASAH2 expression in the liver from 3xTg AD is seen by nine months of age in the 3xTg AD mice compared to NonTg (Figure 3B). Similar to the reported decline in expression of this protein in end-stage AD in humans, 3xTg AD mice with more advanced AD pathology (14-month-old) showed significantly reduced levels of ASAH2 expression in the liver (Figure 3C).

ASAH2 is associated with cell compartmentalization

**Figure 4:**
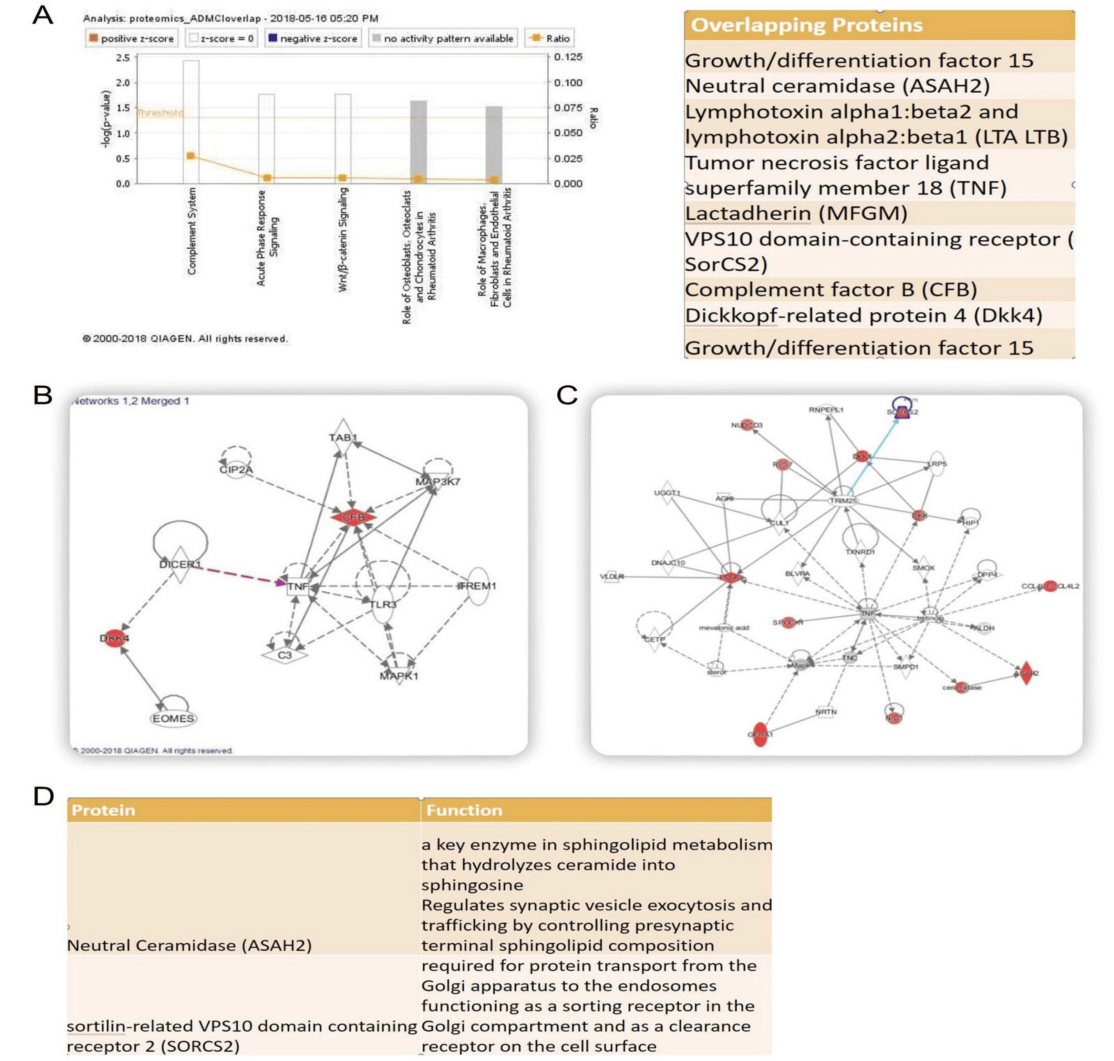
Pathway analysis and protein interaction network of ASAH2. A: Ingenuity pathway analysis (IPA) suggests that molecular pathways involved in inflammation (TNF, complement, lymphotoxin), trafficking (ASAH2 and SORCS2), and growth differentiation (DKK4, GF15) are involved in the initial stages of AD. B, C: Protein-interacting networks are shown for the dysregulated proteins identified on the SOMAscan assay. D: The putative function of ASAH2 and SORCS2 implicates proteins involved in vesicle trafficking for AD. AD: Alzheimer’s disease; ASAH2: N-acylsphingosine amidohydrolase 2

**Figure 5:**
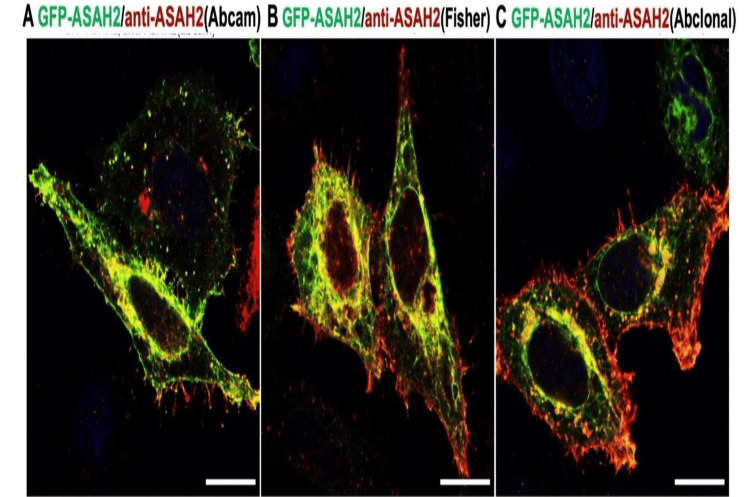
Subcellular localization of ASAH2 in HEK293 human kidney cells. Immunofluorescence images of pc-DNA3-GFP-ASAH2 (green) transfected HEK293 cells with N-acylsphingosine amidohydrolase 2 (ASAH2) antibody (red) purchased from Abcam (A), Fisher (B), and Abclonal (C). Scale bar, 10 mm.

**Figure 6:**
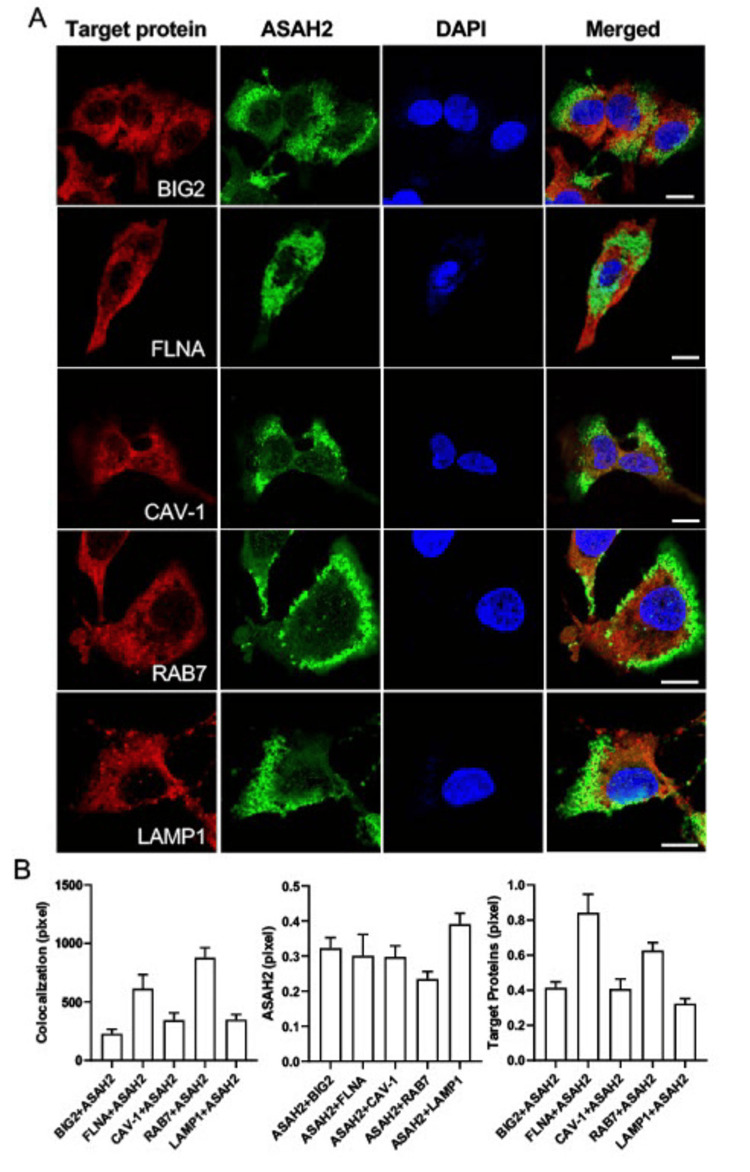
Colocalization of ASAH2 with endolysosome and membrane proteins in human HepG2 liver cells. A: Immunofluorescence images of each of the target proteins (red), N-acylsphingosine amidohydrolase 2 (ASAH2) (green), and DAPI (blue) in HepG2 human liver cells. Target proteins include BIG2, FLNA, CAV-1, RAB7, and LAMP1. Increased overlapping expression is seen between ASAH2 and FLNA, and RAB7. Scale bar, 10 microns. B: Quantification of colocalized immunofluorescence images for the ASAH2 and target proteins in HepG2 human liver cells. The intensity of the ASAH2 and each of the target proteins, including BIG2, FLNA, CAV-1, RAB7, and LAMP1, in the cells was analyzed, respectively (n > 21 cells/group).

IPA suggested several biological clusters were altered in the pre-AD human serum: lipid-associated membrane proteins (i.e., ASAH2, SORCS2, PCSK9), Wnt cascade (DKK4, DKK), and inflammatory pathways (CFB, TNF, lymphotoxin) (Figure 4). ASAH2 is also strongly co-localized in the perinuclear regions in human liver HepG2 and HEK293 cells by immunofluorescence, and to a lesser extent, along the cell membranes, consistent with prior suggestions that this protein is localized to lipid membranes and vesicles (Figures 5, 6). Given prior reports of ASAH2 involvement in caveolin and hepatic endosomes [17,18], we evaluated the subcellular localization of this protein in hepatic cells and its overlapping expression with various membrane- and vesicle-trafficking-associated proteins. We stained the HepG2 cells with each of the target proteins (red), including BIG2, FLNA, CAV-1, Rab7, and LAMP1, with ASAH2 (green) and DAPI (blue), and all stained target proteins were colocalized with ASAH2 (Figure 6A). The overlapping density of the target proteins and ASAH2 was analyzed with the greatest co-localization of either FLNA or RAB7 with ASAH2 (Figure 6B). These findings suggested the associated role of ASAH2 in the membrane and in late endosome to lysosome compartments. 

Reduced endolysosome and membrane protein expressions in the early stage of AD

**Figure 7:**
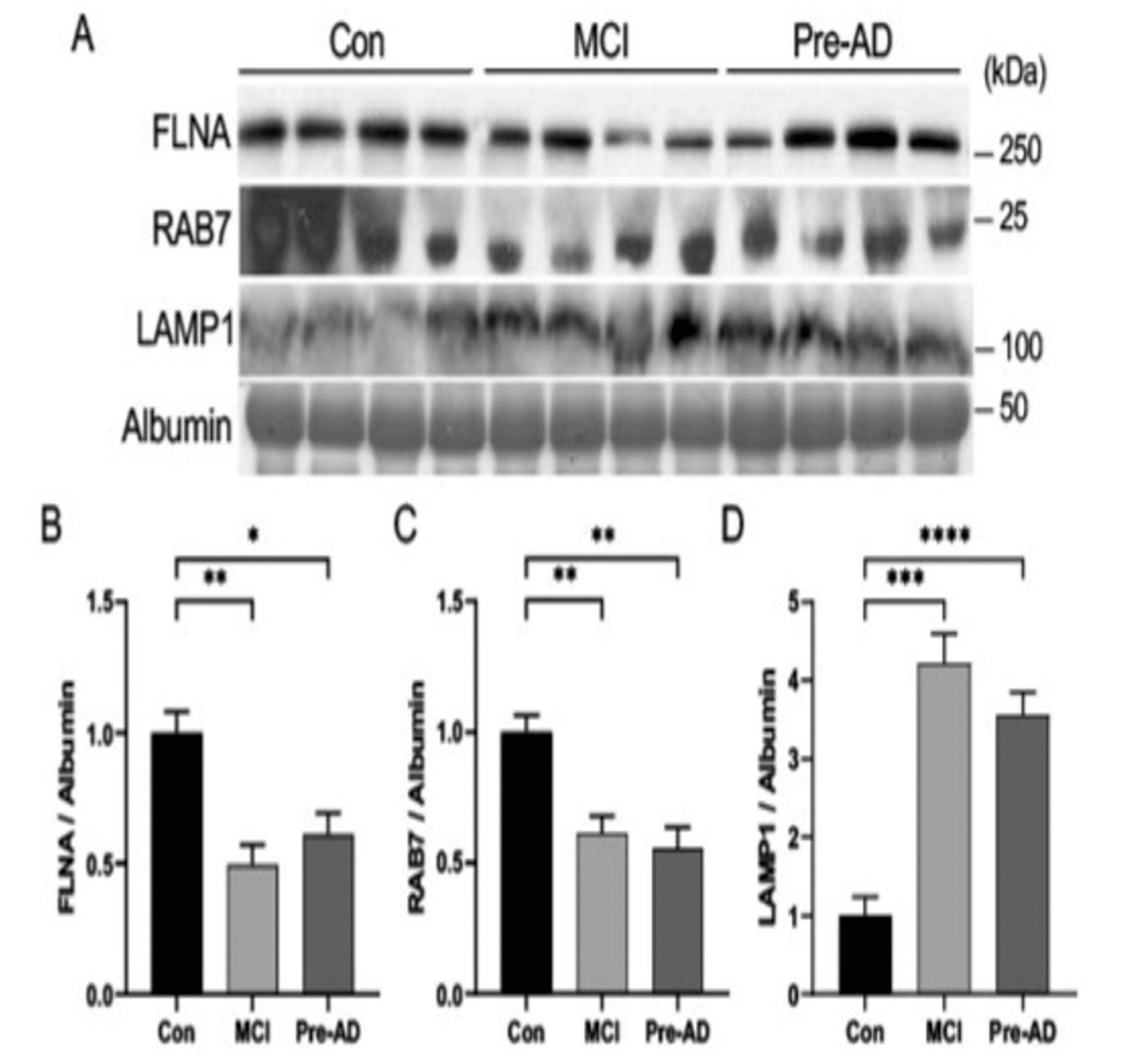
Endolysosome and membrane protein expressions in MCI-, AD-patient, and control human serums. A: Western blot images and the graphs (B-D) of membrane protein and endolysosome protein expression levels in control, MCI-, and AD-patient serums. B, C: The expression levels of membrane protein FLNA (B) and the late endosome transport protein RAB7 (C) were significantly decreased in both MCI- and AD-patient serums compared to those of control human serums. D: Significantly increased level of LAMP1 lysosome protein expression in both MCI- and AD-patient serums compared to that of control human serums. n=4 samples per group. p<0.05, *p<0.01, ***p<0.001, ****p<0.0001, one-way ANOVA (Tukey’s multiple comparison test). AD: Alzheimer’s disease; MCI: mild cognitive impairment; ASAH2: N-acylsphingosine amidohydrolase 2

With the greatest co-localization in the endosome-to-lysosome compartments, we assessed changes in FLNA, RAB7, and LAMP1 in the control, pre-AD, and MCI human serum samples. FLNA and Rab7 protein expressions were significantly decreased compared to Con, whereas the level of lysosome marker, LAMP1, was significantly increased in pre-AD and MCI stage group serums compared to Con (Figure 7). These observations further suggest the involvement of various lipid and membrane-associated proteins in AD onset.

## Discussion

The current study sought to identify serum-derived proteins from individuals with MCI who later progressed to develop AD after five years. We found that the neutral ceramidase ASAH2 and SORCS2 were increased in the affected subjects compared to age-matched controls and individuals with MCI who did not go on to develop AD. ASAH2 has previously been reported to be decreased in AD [16]. We found this same pattern of elevated expression earlier in the AD disease course, followed by progressive and diminished expression levels in late stages of AD, which can be seen in the 3xTg AD mouse model. ASAH2 is broadly expressed in visceral organs and minimally in the brain. Given that ASAH2 is expressed in intracellular vesicles [21,22], we performed co-immunostaining with membrane and vesicle trafficking markers and found the greatest overlap with the late endosomal-lysosomal marker RAB7. Examination of the trafficking-associated markers showed increased LAMP1 and decreased RAB7 and FLNA levels in the MCI and pre-AD subjects. Collectively, these findings implicate several plasma membrane-associated proteins in the serum of pre-AD, MCI patients (in particular, SORCS2, ASAH2, and LAMP1) both as diagnostic markers and as proteins associated with AD progression. Collectively, a subset of plasma membrane-associated proteins may be both important for diagnosis in the early stage of AD and provide insight into pathological mechanisms.

Dysfunction of various trafficking and lipid-dependent signaling pathways is likely involved in the progression of AD neurodegeneration. The SOMAscan profiling yielded two candidate biomarkers, ASAH2/ASAH2B and SORCS2, in MCI and pre-AD subjects. SORCS2 and its family of VPS10p-domain receptors regulate engagement and trafficking of AD-associated proteins between the endosome, Golgi compartments, and the cell surface. All the VPS10p-D receptors (SorLA, Sortilin, SorCS1, SORCS2, and SorCS3) have been identified as AD risk loci [23,24]. We find increased SORCS2 levels in the pre-AD subjects, while VPS10p-D protein expressions are predominantly decreased in AD brains. ASAH2 belongs to a family of neutral ceramidases. Ceramidases regulate the degradation of ceramides into sphingosine. Subsequent phosphorylation of sphingosine produces sphingosine-1-phosphate (S1P). Ceramide has been shown to be involved in stress-related cellular responses and apoptosis, whereas S1P stimulates cell survival, proliferation, and tissue regeneration. Prior work has suggested a susceptibility locus on chromosome 10q11, implicating ASAH2, reduced expression in females across ages, and further reduction in late-onset AD patients.

While our studies identified ASAH2/ASAH2B as a potential biomarker in the pre-AD population, other prior work reported changes in the expression of this ceramidase in late-onset AD. Our finding of this gene's association with AD independently through the SOMAscan proteomic assay and by linkage and candidate gene analyses lends greater credence to its potential role in this neurodegenerative process. It is important to note that these studies were done entirely independently, and the identification of ASAH2 as a marker associated with AD in both our current work and these prior works adds validity to the possible importance of this protein in AD.

ASAH2 functions as a mediator of inflammatory responses. It is upregulated in ulcerative colitis and thought to inhibit inflammation by paradoxically reducing sphingosine and S1P levels, as elevated S1P levels promote leukocytosis. Acute traumatic brain injury (TBI) also leads to increases in ASAH2 levels, but is correlated with an elevation of sphingosine in mitochondria due to the activation of the neural ceramidase and the reduced activity of sphingosine kinase 2. Knockdown of ASAH2 reduces sphingosine accumulation in mitochondria and preserves COX activity (a rate-limiting enzyme in the mitochondrial electron transport chain) after brain injury, thereby improving functional recovery from TBI. Finally, ASAH2 downregulation induces a decrease in cell growth and an increase in neuronal differentiation. Collectively, these studies suggest a role for neutral ceramidase in response to ongoing inflammation.

Characterization of ASAH2 expression suggests an indirect role in AD pathogenesis. Although ASAH2 was first purified from rat brain [25], we do not find high levels of expression of the neutral ceramidase in the brain. Rather in concordance with other studies, high expression levels are appreciated in the adult heart, liver, and gut, endosome-like organelles of hepatocytes, and epithelia of the jejunum and ileum. ASAH2 is an integral membrane protein that can be cleaved to yield a soluble protein and be actively secreted [25,26]. Moreover, ASAH2 is almost completely solubilized by freeze-thawing in both endothelial cells and hepatocytes [26], suggesting that the enzyme is located in intracellular vesicles. We find correlative changes in ASAH2 expression levels in the 3xTg AD mouse liver with progression of the AD phenotype, similar to that seen or reported in humans. In this respect, changes in liver metabolism as reflected through the release of ASAH2 may be an indirect reflection of the AD progression.

The subcellular localization of ASAH2 indicates an integral association with vesicles and, most prominently, the endosomal-lysosomal pathway. Initial characterization studies suggested ASAH2 localization to the Golgi and mitochondria [27]. Later immunofluorescence studies, however, identified mouse ASAH2 mainly in late endosomes/lysosomes, while rat kidney ASAH2 was found to be almost exclusively in lipid rafts of apical membranes. The rapid solubilization and observed secretion of ASAH2 also suggest localization within intracellular lipid membrane vesicles. Consistent with more recent reports, we find the greatest overlapping expression of ASAH2 with the late endosome-/lysosome-associated small GTPase Rab7 and with the more general actin-binding FlnA, which is implicated in endocytosis and vesicle trafficking to the lysosome through formins [28,29]. We also find that expression levels of these proteins are decreased in the pre-AD serum samples. Finally, an increase in the lysosomal LAMP1 protein has been previously reported in pre-AD patients and similarly seen in our studies [30]. Overall, the current studies provide further support for the involvement of sphingolipid metabolism and other vesicle trafficking-associated proteins in AD progression.

Our data strongly implicate an association between AD and ASAH2. The identification of ASAH2 as a potential biomarker using AD samples and Somascan was performed independently of prior reports linking this same protein to end-stage AD. The finding was further supported by changes in ASAH2 expression in the triple transgenic (3x-Tg) AD mouse model. That said, as with all scientific reports, further validation using a larger and independent dataset will be needed to ascertain the significance of our findings.

## Conclusions

This study highlights the potential of ASAH2 and SORC2 as promising biomarkers for AD, providing insights into their characterization at both the tissue and cellular levels. It also highlights the involvement of other vesicle trafficking proteins, such as FLNA, RAB7, and LAMP1, in the pathology of AD. Our findings suggest that alterations in lipid membrane-associated may not be a direct result of central nervous system changes but rather reflect indirect alterations in peripheral organs, particularly the liver. Future research is needed to determine whether these changes are unique to neurodegenerative diseases or more broadly indicative of degenerative and inflammatory conditions.
